# Impact of exosomal HIV-1 Tat expression on the human cellular proteome

**DOI:** 10.18632/oncotarget.27207

**Published:** 2019-09-24

**Authors:** Huafei Lu, Xiaoli Tang, Mitchell Sibley, Jillian Coburn, R. Shyama Prasad Rao, Nagib Ahsan, Bharat Ramratnam

**Affiliations:** ^1^ Division of Infectious Diseases, Department of Medicine, Warren Alpert Medical School, Brown University, Providence, RI 02903, USA; ^2^ COBRE Center for Cancer Research Development, Proteomics Core Facility, Rhode Island Hospital, Providence, RI 02903, USA; ^3^ Biostatistics and Bioinformatics Division, Yenepoya Research Center, Yenepoya University, Mangalore 575018, India; ^4^ Division of Biology and Medicine, Brown University, Providence, RI 02903, USA; ^5^ Clinical Research Center of Lifespan, Providence, RI 02903, USA

**Keywords:** HIV-1, tat protein, exosome, label-free proteomics, ROS

## Abstract

HIV-1 exists in a latent form in all infected patients. When antiretroviral therapy is stopped, viral replication resumes. The HIV-1 Tat protein is a potent activator of viral transcription. Our previous work has demonstrated that exosomal formulations of Tat can reverse HIV-1 latency in primary CD4+ T lymphocytes isolated from long term antiretroviral treated individuals suggesting a potential role for Tat as a therapeutic HIV-1 Latency Reversal Agent (LRA). Here, we employed the label-free proteomic approach for profiling the proteomic changes associated with exosomal Tat production in human cell lines. Comparative proteomic analysis revealed that >30% peptides were differentially expressed in abundance in the Tat-expressing cell line compared with relevant controls. As expected, many of the known Tat-interactor proteins were upregulated. Tat expression also led to the upregulation of antioxidant proteins suggesting Tat-mediates an oxidative burst. Gene ontology and pathway analyses of these differentially expressed proteins showed enrichment of extracellular vesicular exosome and spliceosome localized proteins and proteins involved with transcriptional and translational mechanisms. Our work suggests that HIV-1 Tat expression leads to perturbations in cellular protein expression. *In vivo* administration of Tat using HIV/SIV animal models needs to be performed to assess the physiologic significance of Tat-induced proteomic changes prior to developing HIV-1 Tat as an LRA.

## INTRODUCTION

The HIV-1 Tat protein is a transcription factor with 86 or 101 residues (depending on subtype) that is essential for trans-activating transcription of the HIV-1 viral genome [1, 2]. Tat is encoded by two exons, the first codes for all functional regions and is highly conserved (72 amino acids) and the second exon codes for the variable C-terminal with unknown function. Tat acts by binding to TAR, a structured region in the nascent transcript, and recruits positive transcriptional elongation factor B complex (pTEFb) to the HIV-1 LTR promoter to enhance processive transcription by stimulating the elongation of prematurely terminated transcripts [3]. Without Tat, HIV-1 transcription elongation by RNA Pol II from LTR promoter is very inefficient [4, 5].

In addition to the major role of HIV-1 Tat in the transcriptional activation of HIV viral genome, Tat has been linked to a number of other cellular functions. For example, Tat exposure induces neuronal dysfunction/toxicity and mediates apoptosis in human blood-retinal barrier-associated cells [6, 7]. Tat expression in astrocytes induces astrocyte-mediated neurotoxicity through regulation of MicroRNA-132 [8, 9]. Tat modulates up-regulation of TNF-α expression, and potentiates the TNF- α induced NF-kB activation and cytotoxicity in Hela cells stably transfected with the Tat gene [10, 11]. When treated with SMX-SA, HIV-1 Tat expression in Jurkat cells induced greater level of oxidative stress than the control cell lines by altering the activity of cellular proteins required for homeostasis [12]. These studies indicate that Tat is a multifunctional protein and is involved in multiple cellular activities.

HIV-1 Tat has been implicated in HIV-associated neurocognitive disorder (HAND), a chronic, debilitating condition of variable intensity that appears to be associated with inflammatory and vascular disease markers [13]. Investigators have used comparative proteomics to better define the molecular etiology of HAND vis-a-vis Tat expression. Tat appears to have myriad effects on cellular physiology with perturbations in the expression of proteins and noncoding RNA. For example, a SILAC based comparative proteomics study of neuronal SH-SY5Y cells found that Tat treatment led to the dysregulation of 29 proteins with downregulation of cytoskeletal regulators such as ARHGEF17, SHROOM3 and CMRP1 [14]. Tat treatment of mouse primary glia cells leads to the up-regulation of miRNA-341 which in turn leads to the down regulation of target protein NLRC5 that regulates the NFkB signaling network [15]. Using the SIV (simian immunodeficiency virus) model, investigators have found that uncontrolled viral replication leads to HIV-1 Tat mediated activation of FOXO3 which in turn leads to the down regulation of anti-apoptosis proteins such as gene B-cell lymphoma 2 (Bcl-2) and up-regulation of the pro-apoptosis gene Bcl-2-like 11 [16]. Taken together, these earlier studies clearly showed that Tat not only interacts and/or regulates with its particular interactors but also impacts a diverse set of genes, proteins and metabolites which ultimately regulate many signaling pathways [14, 17–21].

Our interest in the Tat protein derives from recent work in using Tat to activate latent HIV-1. While medical therapy for HIV has improved greatly, the virus persists in CD4+T lymphocytes in a latent state and can rekindle infection when therapy is stopped. This latent reservoir is the major road block to viral eradication. As mentioned, HIV-1 Tat is one of the most potent activators of viral transcription. We have sought to harness this activity and develop Tat as a latency reversal agent (LRA). In a previous study, we demonstrated that Tat could be efficiently loaded into cellular exosomes designed to specifically target CD4+ T lymphocytes (EXO-Tat). Treatment of primary CD4+ T lymphocytes isolated from infected individuals on long term antiretroviral therapy led to latent virus reactivation [22]. Our experiments used HEK293T cells as a factory for the production of EXO-Tat. Here, we profile proteomic changes associated with EXO-Tat production compared to relevant control cells by employing a label-free proteomics platform.

## RESULTS

**Figure 1:**
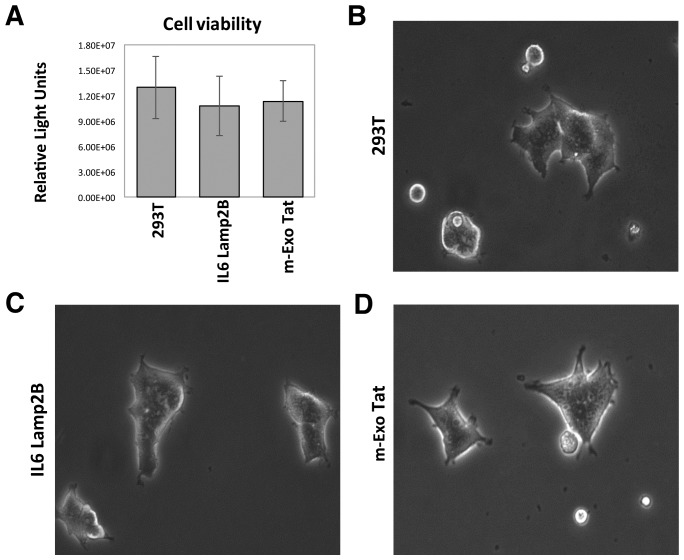
Physiological and morphological characteristics of the 293T and the transfected IL16 Lamp2B and m-EXO Tat cell lines. (**A**) represents the cell viability test and (**B**–**D)** represents the morphological views of the three different cell lines cultured for 72h and visualized under light microscope.

To define whether HIV-1 Tat expression leads to physiological and/or biochemical changes in our experimental cell lines, we first performed a cell viability test which showed no significant differences among the cell lines (Figure 1A). In addition, as expected, there were no visible physiological and morphological changes observed in the Tat overexpressed (EXO-Tat) cell lines compared with the control HEK293T or an empty vector containing cell lines IL16lamp2b (Figure 1B–1D) suggesting that these cell lines are ideal for comparative proteomic analysis.

### Comparative qualitative proteome profiling of Tat-overexpressing cells

**Figure 2:**
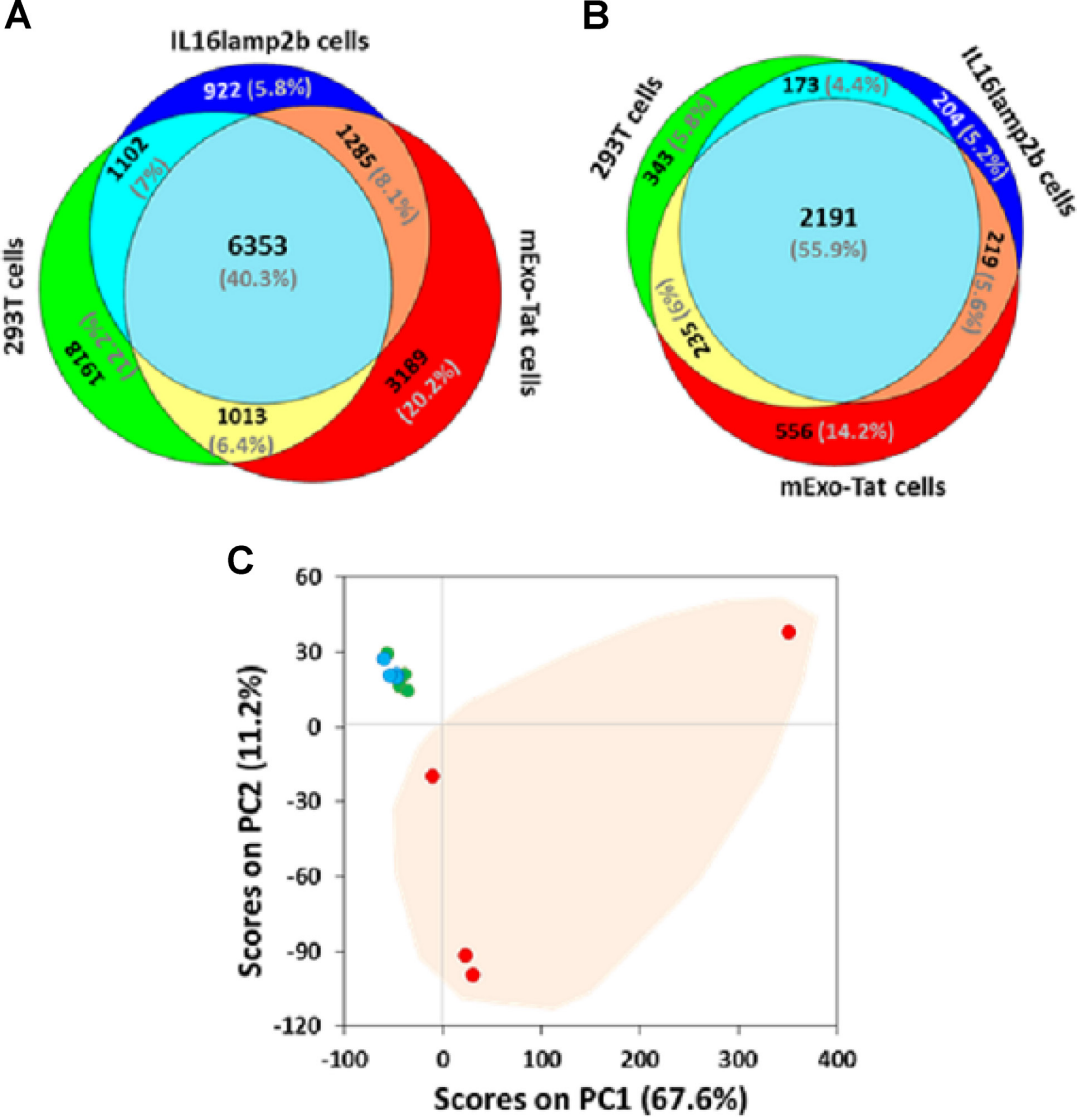
Comparative proteomic profiling of the peptides/proteins identified from 293T, IL16lamp2b and mExo-Tat samples. (**A**–**B)** (the upper panel) show total number of unique peptides and proteins that were common between the various experimental groups. The data are based on any peptides detected in any of the biological replicates for each group. (**C**) principal component analysis (PCA) of peptide abundance data collected from 293T, IL16lamp2b and mExo-Tat samples. Data represents the close clustering of peptide peak areas from 293T cells (green) and IL16lamp2b (blue) and variability in mExo-Tat (red) cells which are distinct from 293T and IL16lamp2b cells.

A comparative label-free proteomic analysis of the three different cell lines showed high reproducibility in terms of proteins/peptides yields across the samples (Supplementary Tables 1–12). For instance, a total of 10386, 9662 and 11840 unique peptides corresponding to 2942, 2787 and 3201 unique proteins were identified from HEK293T, IL16lamp2b (empty vector) and EXO-Tat (mExo-Tat) samples, respectively (Supplementary Table 13). A very similar number of proteins/peptides were identified from each cell line, Venn diagram analysis showed many of the unique and overlapped peptide and proteins in each cell line (Figure 2A–2B). Among the replicates, approximately 40–50% proteins were overlapped in each cell line (Supplementary Figure 1A–1C); wherein 40% of the peptides and 56% of the proteins were overlapped among the cell lines (Figure 2A–2B). Principal component analysis (PCA) was also used to assess similarities and differences between the cell lines as well as the replicates in each cell line. Analyses were performed using four biological replicates per cell line. Results showed discernable differences between the EXO-Tat cell line vs control HEK293T and IL16lamp2b cell lines (Figure 2C). As expected the control HEK293T and IL16lamp2b cell lines were very similar whereas the EXO-Tat cell line was segregated to a greater degree from the control HEK293T and IL16lamp2b cell lines (Figure 2C).

### Quantitative characterization of differentially abundant peptides/proteins

**Figure 3:**
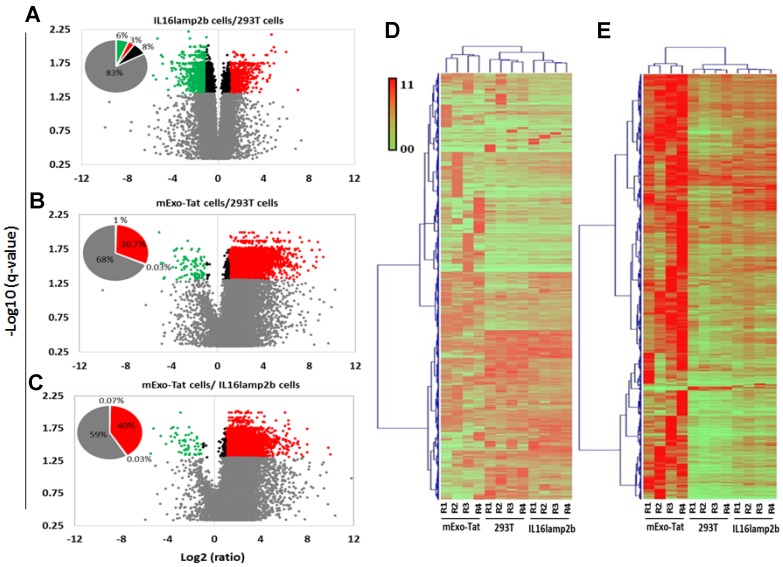
Quantitative analysis of the proteomics dataset obtained from 293T, IL16lamp2b and mExo-Tat samples. (**A**–**C**) volcano plot of fold change versus q-value of peak-area for the total of 19331 peptides quantified. Red and green circles represent the significant (*q* < 0.05) up and down regulated peptides compared with the control 293T cells. Gray circles bellow the blue dot horizontal line (*q* = 0.05) are non-significant and above the lines are significant. Circles between the vertical blue dot lines have 2-fold lower expression. Pie chart shows percentage of the peptide significantly changed compared with the 293T cells. (**D**) HCA and heat map analysis of all peptides and (**E**) significantly changed (*q*-value < 0.05, at least 2 fold differences) peptides show clustering based on 293T, IL16lamp2b and mExo-Tat cell lines. Peptide expression of mExo-Tat cell lines being distinct from the other two cell lines and shows much more variability. Most of the significantly changed peptides in mExo-Tat cell lines are up-regulated. Heat map shows scaled peak area for peptides.

A peak area based relative quantitative analysis was performed among the identified peptides between the various groups. A total of 19331 peptides corresponding to 3138 unique proteins were subjected for quantitative analysis (Supplementary Table 14) wherein a total of 8506 unique peptides corresponding to 2436 showed changes in peptide abundance in at least one condition. The distribution of significant peptides based on fold-differences in abundance of three cell populations showed as volcano plots (Figure 3). As expected, a very low number of peptides (9%) were significantly different in abundance (>2-fold) when compared between HEK293T and IL16lamp2b cell lines (Figure 3A). Conversely, 31–41% of peptides in HEK293T and IL16lamp2b cell lines revealed relative fold change greater than 2 (*q* < 0.05) compared to EXO-Tat cells, wherein the majority of the differentially expressed peptides exhibiting a higher peak area in EXO-Tat cells indicating that the majority of peptides was upregulated compared with the other two control cell lines (Figure 3B–3C).

For better visualization and further validation of above observations including the PCA and volcano plot analyses, heat maps were constructed to illustrate the clustering of all identified peptides (Figure 3D) and the significantly differentially expressed peptides in each of the three replicates of HEK293T, IL16lamp2b and EXO-Tat cell lines (Figure 3E). Heat map analysis revealed an overall similar pattern of peak-area quantitation, with many of the proteins and peptide sequences within HEK293T and IL16lamp2b cell lines wherein peptide/proteins expression in EXO-Tat cell lines exhibiting mostly increased in abundance compared with the other two control cell lines. Taken together, these quantitative analysis results allowed us to conclude that there was very minimal significant difference between HEK293T and IL16lamp2b cell populations however, proteins/peptides expression pattern is very different in EXO-Tat cell lines.

### Functional classification and pathway analysis of differentially accumulated proteins

**Figure 4:**
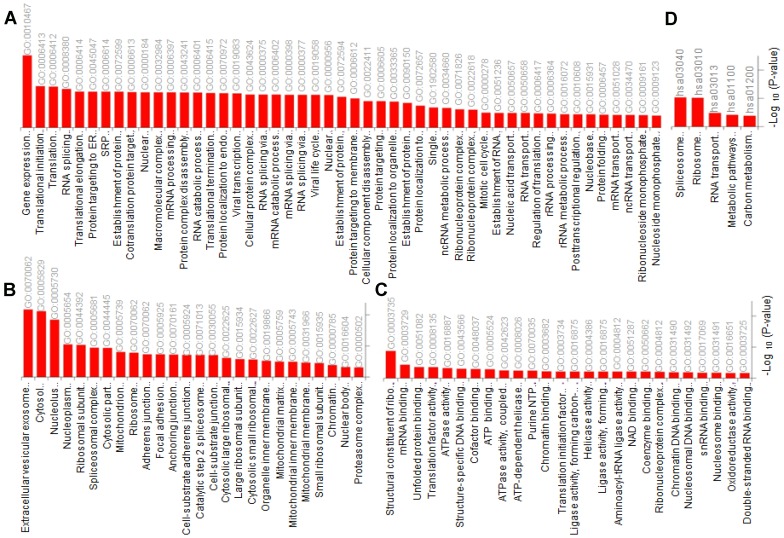
Gene ontology (GO) analysis using DAVID (https://david.ncifcrf.gov/). Proteins with differential expression in 293T cells versus mExo-Tat cells showed enrichment (Bonferroni corrected *P*-value < 0.05) for (**A**) biological process, (**B**) cellular component (**C**) molecular function and (**D**) KEGG pathways.

For better understanding of the affected pathways in Tat transfected cell lines, peptides/proteins with significant difference in abundance in EXO-Tat cell lines compared with the control HEK293T were further subjected to gene ontology analysis followed by pathway analysis using Enricher (Figure 4). Gene ontology analysis showed gene expression and translational pathways highly enriched in the biological process category (Figure 4A) wherein proteins localized in extracellular vesicular exosome, cytosol and nucleolus are enriched in cellular component category (Figure 4B). Similar with the biological process, proteins associated with structural constituent of ribosome (GO:0003735), mRNA binding (GO:0003729) and unfolded protein binding (GO:0051082) are enriched in molecular function category (Figure 4C). The gene ontology data is further supported by the KEGG pathway analysis wherein the Spliceosome (hsa03040) and ribosome (hsa03010) pathways are mostly enriched (Figure 4D). It is important to note that proteins listed in both of these pathways are associated predominantly in post-transcriptional mechanisms suggesting that the translational machinery is significantly affected in EXO-Tat cell lines.

### Identification and expression of known Tat-interactor proteins in EXO-Tat cell lines

**Figure 5:**
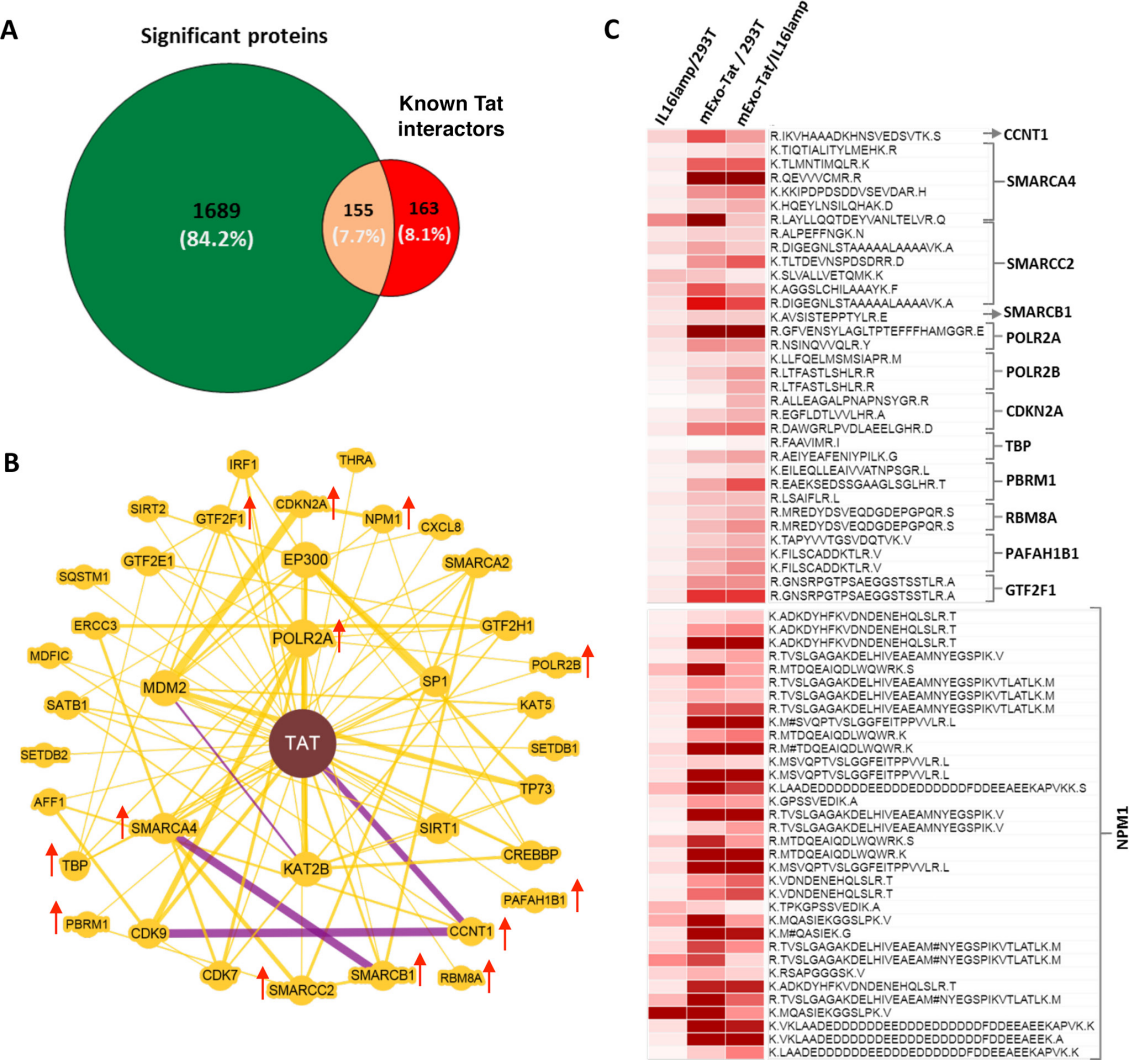
Identification and differential expression of known Tat-interactors. (**A**) shows the number of the known Tat interactors overlapped with the proteins with significant changes in abundance in mExo-Tat cells compared with the other two cell lines. A total of 318 proteins are extracted from BioGRID (https://thebiogrid.org/1205541) and IntAct (https://www.ebi.ac.uk/intact/interactors/id:P04608) database and as Tat interactor. (**B**) represents the Tat_human protein-protein interaction map generated by BioGRID wherein a total of 37 proteins with known interactions with Tat. Greater node size represents increased connectivity and thicker edge sizes represent increased supporting association. Purple edge represents both genetic and physical evidence wherein yellow edge represents only physical evidence. Red arrow indicates those proteins identified in this study and increased in abundance in mExo-Tat cell lines over the controls. (**C**) shows the heat map analysis of the fold change of the peptide corresponding to the identified Tat interactors in (B).

**Figure 6:**
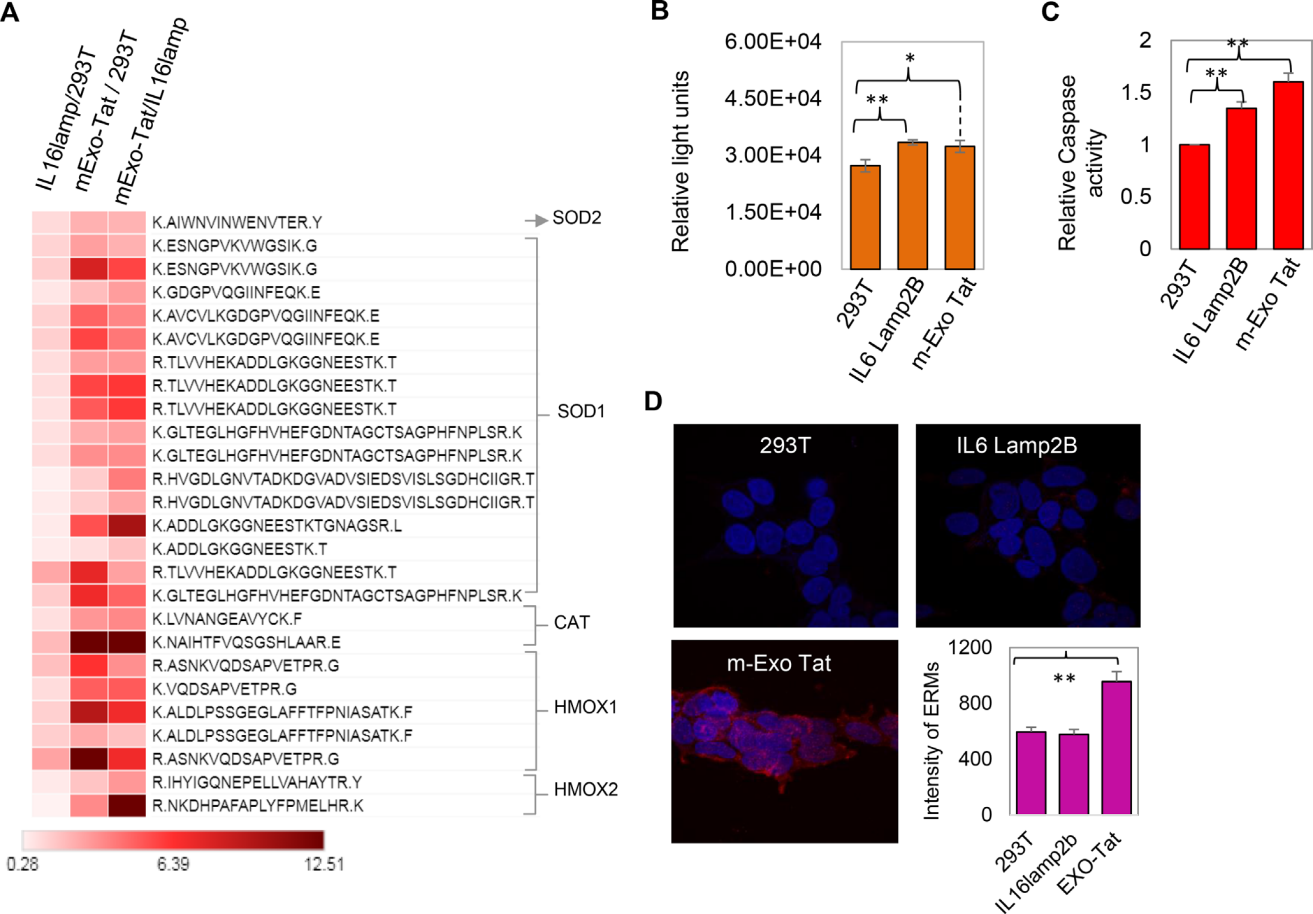
Biochemical characteristics of the 293T and the transfected IL16 Lamp2B and m-EXO Tat cell lines. (**A**) the heat map analysis of the fold change of the peptides corresponding to the antioxidant proteins. (**B**–**C**) reactive oxygen species and caspase activity assay. (**D**) represents the ERM protein labeling assay. For ERM protein measurements, 293T, IL16lamp2b and EXO-Tat cells were prepared and probed with ERM antibodies. Positive staining was defined through intensity thresholding for mean intensity measurements. A total of twelve images per samples were acquired with the same settings and analyzed. All data represents the average and standard deviation values from three separate replicate experiments. For all panels, unpaired t-test analysis was used (^*^ *P* = 0.01–0.05; ^**^ *P* = 0.003).

We further demonstrated the expression pattern of the known Tat-interactor proteins identified in this study. A total of 318 proteins are listed in publicly accessible databases such as BioGRID (https://thebiogrid.org/1205541) and IntAct (https://www.ebi.ac.uk/intact/interactors/id:P04608) known as potential Tat-interactor proteins. A comparative analysis showed that approximately 50% (155) of the Tat-interactor proteins were overlapped with our significantly changed proteins (Figure 5A). In addition, a protein-protein interaction map generated with BioGRID using Tat and 37 Tat-interacting human proteins showed 35% of the proteins were up-regulated in EXO-Tat cell lines compared with the controls (Figure 5B). The number of peptides identified corresponding to those Tat-interactor proteins are highly variable (1–34), however the expression pattern is very similar in EXO-Tat cell lines (Figure 5C). Except in a few peptides, the fold change ratio of most of the peptides were much higher from 2 fold to up to 35 fold in EXO-Tat vs HEK293T and EXO-Tat vs IL16lamp2b cells when compared with the Il16lamp2b vs HEK293T cell lines. As shown in Figure 6B–6C, most of the studied Tat-interactors such as CCNT1, SMARCA4, SMARCC2, SMARCB1, POLR2A, POLR2B TBP, NPMM1 were successfully identified and showed increased abundance in the EXO-Tat cell lines indicating that overexpression of Tat positively regulates its potential interactors.

## DISCUSSION

The HIV-1 protein Tat has the potential to be harnessed to reverse HIV-1 latency. The major clinical challenge will be to deliver Tat to HIV-1 infected CD4+ T lymphocytes *in vivo*. We have therefore designed an exosomal platform to allow *in vivo* Tat delivery. Components of this platform include the genetic modification of Tat and its inclusion in a vector (pEXO-Tat) that specifically packages Tat in cellular exosomes. We introduced a further genetic manipulation to target exosomes to CD4+ T lymphocytes (IL16lamp2b cells). Our long term plan is to package EXO-tat on a recombinant viral vector to allow for its production in the infected host. This study was conducted to measure the proteomic sequelae of exosomal Tat production in HEK293 T cells. Control cells included both wild type HEK293T cells and cells that had been transfected with a recombinant vector to redirect exosomes to CD4+ T lymphocytes (IL16lamp2b cells). Approximately 30% of the mExo-Tat cell lines expressed Tat **(** Supplementary Figure 4) which might be responsible for the minimal visible physiological and morphological changes in the mExo-Tat cell lines. However, we hypothesized that dissecting of proteome profiles coupled with label-free quantitative analysis would be one of the best options for demonstrating the global impact of Tat expression in human cells.

Differential expression of genes and proteins has been demonstrated in various cell lines overexpressing Tat protein [14, 18–21]. In addition, a recent report showed the dysregulation of the proteome of human CD4^+^ T cells in response to HIV-1 infection both *in vitro* and *in vivo* [23]. However, in this report, for the first time we have demonstrated how expression of the HIV-Tat protein in exosomes could interrupt the cellular proteome wherein at least 30% of the identified peptides associated with translational machinery and metabolic pathways are predominantly increased in Tat-overexpressing cells. These findings are in good agreement with the metabolomic analysis conducted by Liao *et al.* [19] on Jurkat cells wherein approximately 70% of the metabolites associated with11 cellular pathways were significantly increased by Tat treatment which was further supported by gene expression analysis of 10 relevant metabolite enzymes [19]. Furthermore, we evaluated our proteomics results with other published datasets wherein Tat-interactors and Tat-regulated proteins were identified using affinity purification and Tat- and HIV-1 treated/infected cells, respectively, followed by LC-MS/MS analysis (Supplementary Figure 2).

As shown, the numbers of differentially expressed proteins are much higher in our study compared with other published datasets which could be associated with higher number of proteins/peptides identified by using a more sensitive mass spectrometer instrument followed by the label-free quantitation method used in this study as well as the use of stable Tat overexpressing cell lines. However, it is important to note that the numbers of overlapping proteins compared with other proteomic studies ranged from 25–67% (Supplementary Figure 2) indicating the reliability of the Tat-regulated proteins in cells. The highest 67% corresponding to 75 differentially expressed proteins overlapped with our study and Navare *et al.* [24], wherein CD4^+^ lymphoblastoid SUP-T1cells were infected with HIV-1 strain LAI. Similarly, a total of 230 proteins corresponding to 58% of the differentially expressed protein were overlapped with Ganief *et al.* (2017). This comparative proteome analysis revealed that other than the known Tat-interactors, a total of 28 proteins were overlapped with multiple individual studies (Supplementary Figure 2). Together with some ribosomal proteins, ACP1, ABCF1, AKR1B1, CFL1, EEF1B2, EIF2S1, EIF3K, HMGB1, LDHA, MAP2K2, NUDC, PCNA, SET, SLC3A2, SUB1, TFRC and XRCC6 were identified in at least three different studies suggesting that these proteins could be used as potential Tat/HIV-1 regulated protein markers in human cell lines. Interestingly, a protein-protein network showed 79% of these proteins are tightly connected to each other and co-expressed and experimentally determined to interact with each other (Supplementary Figure 3).

For instance, EEF1B2 (Eukaryotic translation elongation factor 1 beta 2), EIF2S1 (Eukaryotic translation initiation factor 2, subunit 1 alpha), EIF3K (Eukaryotic translation initiation factor 3, subunit K), ABCF1 (ATP-binding cassette, sub-family F), SUB1 and SLC3A2 (Solute carrier family 3, member 2) are interconnected with single or multiple ribosomal proteins thus regulating protein translation. Differential expression of these proteins in response to HIV-1 and/or Tat exposure has also been reported in multiple studies [14, 17, 23–25]. Similarly, many metabolic enzymes including LDHA with their corresponding metabolites have been increased significantly in human cell lines upon exposure to HIV-1 Tat protein [14, 19, 24]. Our study shows a positive connection between the metabolic enzymes followed by increasing of several metabolites and the cytoskeleton dynamic associated proteins such as CFL1, PFN1, CAP1 in response to HIV-1/Tat treatment indicating immune and cellular structural remodeling to cope with the toxicity [19]. GO analysis of these proteins showed expected results such as in case of biological and molecular process, the highest numbers of proteins are associated with translational initiation and nucleic acid binding (Supplementary Figure 3).

Although, generation of ROS or oxidative stress in HIV patients is thought to be one of the major characteristics [26], there is very little evidence at the proteome level so far to support this hypothesis. The comparative proteomic analysis between HEK293T and EXO-Tat cell lines revealed increased peptide abundance of many known first line antioxidant proteins such as SOD1, SOD2, CAT, HMOX1 and HMOX2 in EXO-Tat cell line indicating a positive correlation of Tat expression with oxidative stress in cells (Figure 6A). Consistent with our observation, mRNA expression levels of many antioxidant genes including SODs, CAT and HMOX were found to be increased along with the gradual increase of cellular H_2_O_2_ level in the Tat treated human neuroblastoma cells (SH-SY5Y) [21].

To further validate the fundamental question of whether Tat-overexpression altered the ROS levels in cells, we measured the cellular ROS levels. Interestingly, ROS was significantly increased in both IL6Lamp2B and mExo-Tat cells compared with the HEK293T cells (Figure 6B). Together with the ROS, apoptosis assay measured by caspase activity also increased in both IL6Lamp2B and mExo-Tat cells (Figure 6C), however, ERM (ezrin/radixin/moesin) protein assay indicates that overexpression of Tat significantly altered the cellular dynamics in mExo-Tat cells compared with the HEK293T and IL6Lamo2B cell lines (Figure 6D). Increased ROS production and caspase activity in the IL6Lamp2B cell lines compared to the HEK293T could be due to the effect of transfection agents and/or methods [27]. Transfection agents and method mediated alteration of gene expression and cellular cytotoxicity has been reported in many studies [27–29].

Although comparative proteomic analysis reveals alterations of many proteins in the IL6Lamp2B cell line when compared with the HEK293T cells, the overall protein expression pattern in HEK293T and IL6Lamp2B cell lines are comparable (Figures 2C and 3E). Therefore, very similar numbers of peptides/proteins were significantly altered in the HEK293T and IL6Lamp2B cell lines when compared with the mExo-Tat cells (Figure 3A). It is important to note that, in this study, the Tat-induced differential protein expression was considered when a protein was statistically significantly altered in abundance in mExo-Tat cells compared to the HEK293T and IL6Lamo2B cell lines.

The potential use of HIV-1 Tat as a therapeutic has been largely investigated in vaccinology with administration of Tat antigen being well tolerated by human subjects [30]. The potential effect of continuous Tat expression *in vivo* has not been studied from a proteomic angle. Our comparative proteomic results point to wide perturbations in cellular processes such as the transcriptional and translational machinery and protein targeting. As expected, known-Tat interacting proteins were identified. Unexpectedly, Tat expression was associated with up-regulation of proteins associated with oxidative stress and apoptosis. The further clinical development of HIV-1 Tat as an LRA will require better understanding of proteomic changes following Tat administration *in vivo* using models such as humanized mice or macaques.

## MATERIALS AND METHODS

### Development of HIV-1Tat overexpressing cell line and cell culture

HEK293T cells were cultured in Dulbecco’s modified Eagle’s medium (Life Technologies) with 10% fetal bovine serum (FBS) (Thermo Scientific, USA), 2 mM l-glutamine and non-essential amino acids (Life Technologies). The cells were trypsinized and reseeded in culture plates 1day before transfection. HEK293T cells were transfected with lipofectamine when cell confluency was ~70%. Stable cell lines were developed by infecting HEK293T cells with lentiviruses encoding EXO-Tat or IL16Lamp2b as described previously [22]. Briefly, EXO^CD4^-Tat stable cells refer to a cell line that exports HIV-1 Tat in cellular exosomes that target the human CD4 receptor. The cDNA fragment encoding HIV-1 Tat protein with a myc nuclear localization signal fused to its C-terminus was sub-cloned into XPack CMV-XP-MCS-EF1-Puro Cloning lentivector. The generated construct was named pEXO-Tat. A lentiviral packaging plasmid pPACKH1 (System Biosciences) was co-transfected into HEK293T cells with pEXO-Tat at the ratio 2:1 to generate EXO-Tat lentiviruses. Stable EXO-Tat cells were established by transducing HEK293T cells with the EXO-Tat lentiviruses at MOI of 10 under the pressure of puromycin. CD4T receptor targeting was accomplished by transducing EXO-Tat stable cells with the IL16lamp2b lentiviruses at MOI of 10 under the pressure of puromycin, as noted below. We named the resulting cell line as EXO^CD4^-Tat

EXO^CD4^ refers to cells that produce exosomes that target the human CD4 receptor. The cDNA fragment encoding the C-terminal domain of interleukin 16 fused to the N-terminus of lysosome-associated membrane protein 2 variant b (lamp2b) was cloned into pCDH-EF1-MCS-T2A-Puro (System Biosciences) Cloning lentivector. The generated construct was named pIL16lamp2b. A lentiviral packaging plasmid pPACKH1 was cotransfected into HEK293T cells with a pIL16lamp2b plasmid at the ratio 2:1 to generate IL16lamp2b lentiviruses. Stable IL16lamp2b cells were established by transducing HEK293T cells with the IL16lamp2b lentiviruses at MOI of 10 under the pressure of puromycin.

### Cell viability and reactive oxygen species assays

Cell lines HEK293T, IL16lamp2b and EXO-Tat were cultured in Dulbecco’s Modified Eagle medium (GE Healthcare Bio-Sciences, PA, USA) with 10,000 U/mL penicillin-streptomycin (Thermo-Fisher, USA), 200 mM of L-Glutamine (Thermo-Fisher, USA), and fetal bovine serum over a period of 48 hours. Each of the cell lines were cultured in five plates and considered as five replicates. After dissociation with 0.25% trypsin (Thermo-Fisher, USA), cells were centrifuged and re-suspended in PBS buffer (GE Healthcare Bio-Sciences, PA, USA). An equal number of cell (3.0 × 105/mL) concentrations were used for each assay.

Cell Viability assay was preformed using the CellTiter-Glo 2.0^®^ bioluminescence assay kit (Promega, WI, USA) according to the manufacturer’s protocol. Readings were taken using the Glo-Max Explorer system (Promega, WI, USA), wherein higher luminescence correlated to viability [31]. Readings were taken using the Glo-Max with higher luminescence correlating to more cell death [32]. Reactive Oxygen Species (ROS) were measured using ROS-Glo™ H_2_O_2_ assay kit (Promega, WI, USA) according to the manufacturer’s protocol wherein higher signals are indicative of increased amount of ROS in cells [33].

### Sample preparation for proteomic analysis

Pellets from HEK293T, IL16-lamp2b and EXO-Tat cell lines (four replicates/cell line) were subjected to a lysis buffer (8 M urea, 1 mM sodium orthovanadate, 20 mM HEPES, 2.5 mM sodium pyrophosphate, 1 mM β-glycerophosphate, pH 8.0, 20 min, 4°C) followed by a 30 sec sonication. Protein extraction, trypsin digestion and tryptic peptide de-saltation were performed as described previously [34]. Briefly, the cleared supernatant was collected by centrifugation at 14 000 × g for 15 min at 4°C. Protein concentration was measured by BCA kit (Pierce BCA protein assay, Thermo Fisher Scientific, IL, USA). A total of 100 μg of protein per sample was subjected for trypsin digestion. Tryptic peptides were desalted using C_18_ Sep-Pak plus cartridges (Waters, Milford, MA) and were lyophilized for 48 hours to dryness. The dried eluted peptides were reconstituted in buffer A (0.1 M acetic acid) at a concentration of 1 μg/μl and 5 μl was injected for each analysis.

### LC-MS/MS analysis

The LC-MS/MS was performed on a fully automated proteomic technology platform that includes an Agilent 1200 Series Quaternary HPLC system (Agilent Technologies, Santa Clara, CA) connected to a Q Exactive Plus mass spectrometer (Thermo Fisher Scientific, Waltham, MA). The LC-MS/MS set up was used as described earlier [35]. Briefly, the peptides were separated through a linear reversed-phase 90 min gradient from 0% to 40% buffer B (0.1 M acetic acid in acetonitrile) at a flow rate of 3 μl /min through a 3 μm 20 cm C_18_ column. The electrospray voltage of 2.0 kV was applied in a split flow configuration, and spectra were collected using a top-9 data-dependent method. Survey full scan MS spectra (*m/z* 400-1800) were acquired at a resolution of 70,000 with an AGC target value of 3 × 10^6^ ions or a maximum ion injection time of 200 ms. The peptide fragmentation was performed via higher-energy collision dissociation with the energy set at 28 NCE. The MS/MS spectra were acquired at a resolution of 17,500, with a targeted value of 2 × 10^4^ ions or a maximum integration time of 200 ms. The ion selection abundance threshold was set at 8.0 × 10^2^ with charge state exclusion of unassigned and *z* =1, or 6–8 ions and dynamic exclusion time of 30 seconds.

### Database searching

Peptide spectrum matching of MS/MS spectra of each file was searched against the Human UniProt database (UniProt; downloaded 2/1/2015) using MASCOT v. 2.4 (Matrix Science, Ltd, London, UK). A concatenated database containing “target” and “decoy” sequences was employed to estimate the false discovery rate (FDR) [36]. Msconvert from ProteoWizard (v. 3.0.5047), using default parameters and with the MS2Deisotope filter on, was employed to create peak lists for Mascot. The Mascot database search was performed with the following parameters: trypsin enzyme cleavage specificity, 2 possible missed cleavages, 10 ppm mass tolerance for precursor ions, 20 mmu mass tolerance for fragment ions. Search parameters permitted variable modification of methionine oxidation (+15.9949 Da) and static modification of carbamidomethylation (+57.0215 Da) on cysteine. The resulting peptide spectrum matches (PSMs) were reduced to sets of unique PSMs by eliminating lower scoring duplicates. To provide high confidence, the Mascot results were filtered for Mowse Score (>20). Peptide assignments from the database search were filtered down to a 1% FDR by a logistic spectral score as previously described [36, 37].

### Relative quantitation of the identified peptides

Relative quantification of peptide abundance was performed via calculation of selected ion chromatogram (SIC) peak areas. Retention time alignment of individual replicate analyses was performed as previously described [35]. Peak areas were calculated by inspection of SICs using in-house software programmed in R 3.0 based on the Scripps Center for Metabolomics’ XCMS package (version 1.40.0). This approach performed multiple passes through XCMS’ central wavelet transformation algorithm (implemented in the centWave function) over increasingly narrower ranges of peak widths, and used the following parameters: mass window of 10 ppm, minimum peak widths ranging from 2 to 20 seconds, maximum peak width of 80 seconds, signal to noise threshold of 10 and detection of peak limits via descent on the non-transformed data enabled. SIC peak areas were determined for every peptide that was identified by MS/MS. In the case of a missing MS/MS for a particular peptide, in a particular replicate, the SIC peak area was calculated according to the peptide’s isolated mass and the retention time calculated from retention time alignment. A minimum SIC peak area equivalent to the typical spectral noise level of 1000 was required of all data reported for label-free quantitation. Individual SIC peak areas were normalized to the peak area of the standard synthetic peptide DRVYHPF that was exogenously spiked prior to reversed-phase elution into the mass spectrometer. Quantitative analysis was applied to replicate experiments. To select peptides that show a statistically significant change in abundance between control vs treatment cells, *q*-values for multiple hypothesis tests were calculated based on *p*-values from two-tailed unpaired Student’s *t* tests using the R package QVALUE as previously described [38, 39].

### Bioinformatics analyses

Venn diagrams were prepared using freeware from the Pacific Northwest National Laboratory (http://omics.pnl.gov/software/venn-diagram-plotter) and VENNY 2.0 (bioinfogp. cnb. csic. es/tools/venny/). Volcano plots used adjusted *p*-values derived from pair-wise ANOVA results and fold-change for peptide peak areas. Principal component analysis (PCA) and heat map/hierarchical clustering were done in R. Cytoscape was used for Network Visualization. Gene ontology (GO) analysis was done using Enrichr (http://amp.pharm.mssm.edu/Enrichr/) and DAVID (https://david.ncifcrf.gov/).

### Apoptosis assay

Apoptosis assay was performed using Caspase Glo 3/7 assay kit (Promega, WI, USA) according to the manufacturer’s protocol. Briefly, 293T, IL16lamp2b and EXO-Tat cells were grown in 96-well plates at the same density for 48 hours and added with Caspase Glo 3/7 reagent and the luminescence measured in a plate-reading luminometer, wherein the luminescence is proportional to the amount of caspase activity.

### Immunocytochemistry

The 293T, IL16lamp2b, and EXO-Tat cells were grown on slides for 48 hours under the above mentioned optimized conditions. Cells were then fixed in 4% paraformaldehyde for 10 minutes followed by 3× washing in PBS and subsequently blocked in normal goat serum (Sigma Aldrich, USA) for 1 hour at room temperature. Cells were stained with mouse HA mAb and/or rabbit ERM pAb (Cell signaling Technology, USA) overnight and washed 3 times with PBS. Cells were incubated with Alexa Fluor 594 goat anti-mouse or anti-rabbit IgG (Invitrogen, USA) for 1 hour followed by 3× washing with PBS. For HA-tagged IL16lamp2b and EXO-Tat proteins, fifteen fields per slide were randomly selected and each channel was acquired separately. HA positive staining was defined through intensity thresholding and percent HA positive cells determined. For ERM proteins, twelve-bit grayscale images were acquired per specimen with a Nikon E800 microscope (Nikon Inc. Melville NY) using a 40× Plan Apo objective and a Spot RT3 camera. Camera settings were based on the brightest slide and the cameras built-in green filter was used to increase image contrast. (Diagnostic Instruments, Sterling Heights MI). All subsequent images were acquired with the same settings. Image processing and analysis were performed using iVision image analysis software. Positive staining was defined through intensity thresholding for mean intensity measurements.

## SUPPLEMENTARY MATERIALS




























