# Supplementary material for: Impact of exosomal HIV-1 Tat expression on the human cellular proteome
Source: Oncotarget. 2019 Sep 24;10(54):5632–44. doi: 10.18632/oncotarget.27207 (PMC6771461; doi:10.18632/oncotarget.27207)
Supplement: Supplementary file 1 [file oncotarget-10-5632-s001.pdf]

Impact of exosomal HIV-1 Tat expression on the human cellular proteome

SUPPLEMENTARY MATERIALS

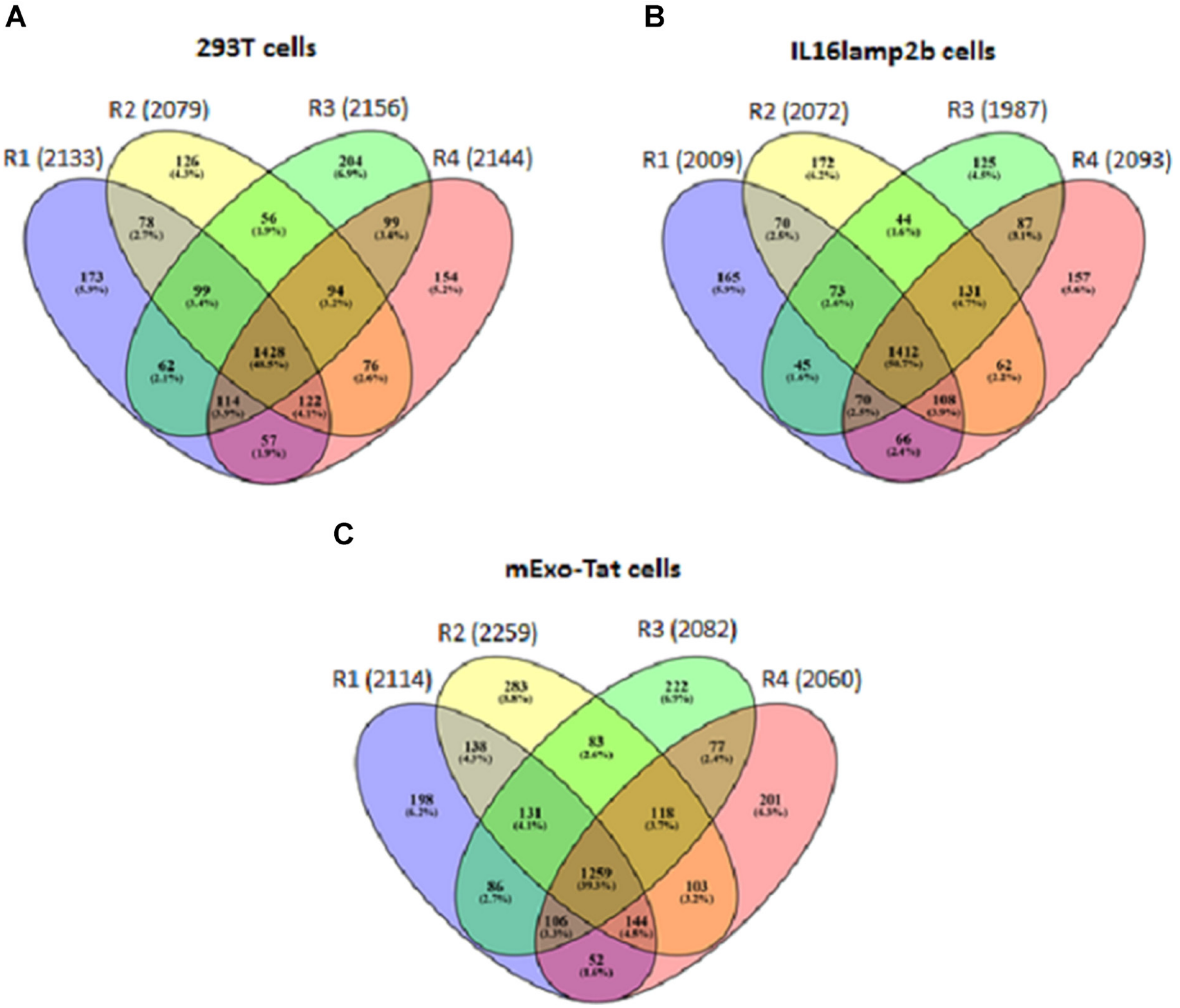

Supplementary Figure 1: Venn diagrams showing the detection of proteins and peptides across biological replicates for each experimental group. (**A–C**) shows the four biological replicates for each experimental group. The data are based on any peptides detected in any of the biological replicates for each group.

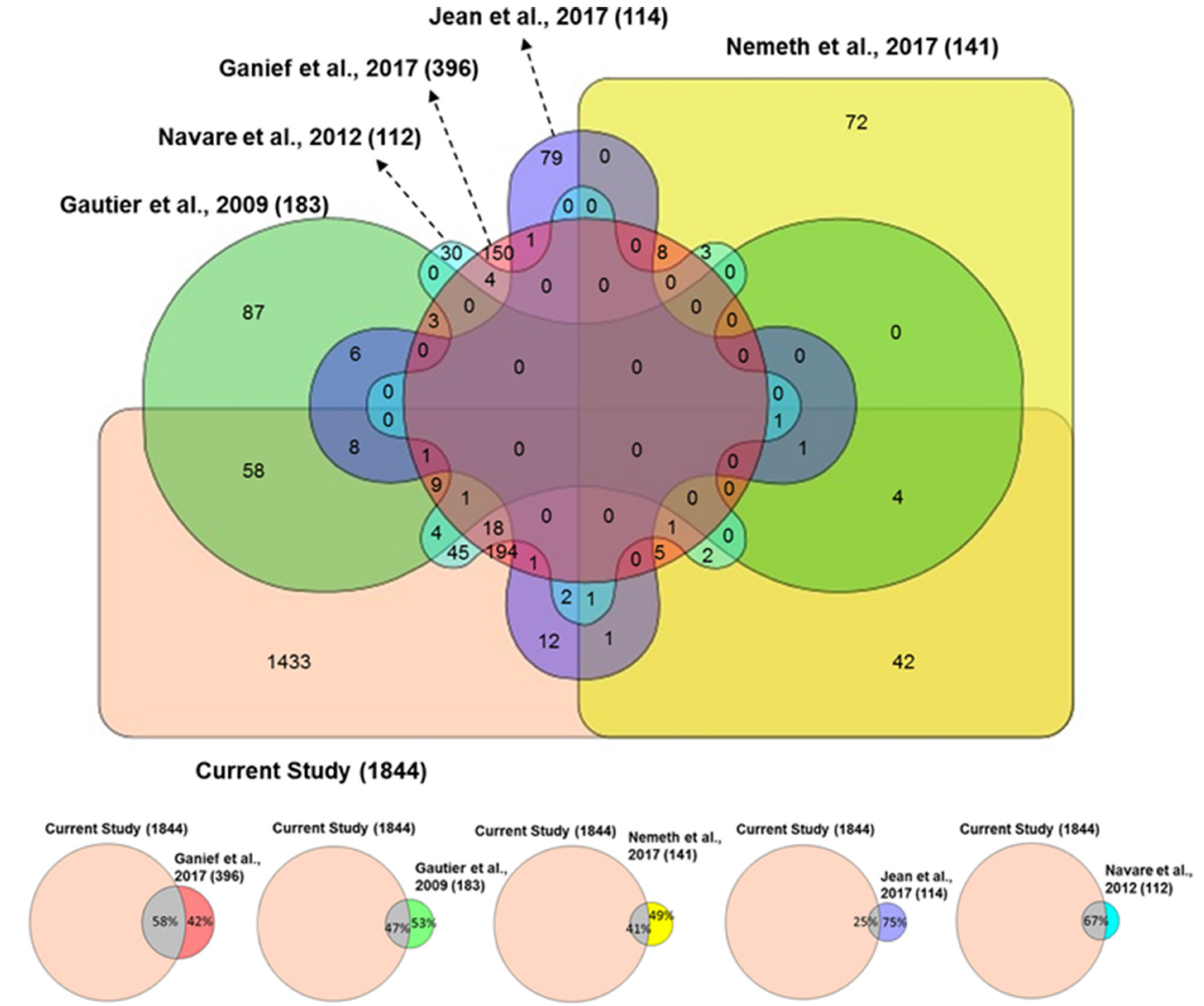

Supplementary Figure 2: Comparative analysis of Tat/HIV-1-induced and/or regulated proteins identified in human cell lines in various independent studies. The upper panel shows the Venn diagram analysis of the differentially expressed proteins in six individual studies including this study wherein cells were overexpressing Tat and/or exposed to HIV infection or Tat protein. The lower panel shows the proportional Venn diagram analysis of the percentage of differentially expressed proteins of other individual studies overlapping with the current study. The gray color in the proportional Venn diagram indicates the overlapping proteins.

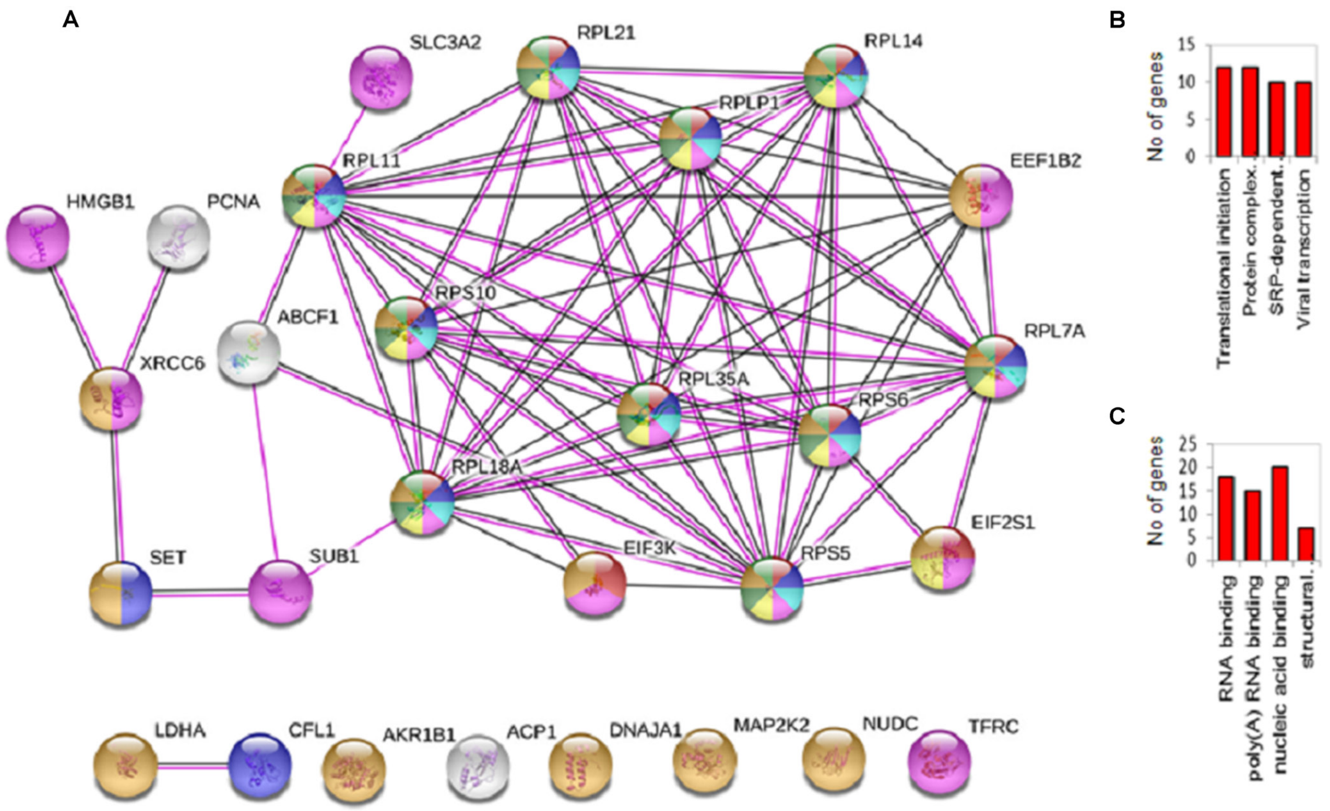

Supplementary Figure 3: Protein-Protein interaction (PPI) networks of the Tat/HIV induced proteins common in at least three individual studies. (**A**) represents the protein-protein networks with a total of 28 common proteins (nodes) connecting with 71 edges. The PPI enrichment *p*-value is < 1.0e-16. The nodes colors represent the following information: red, translational initiation; blue protein complex disassembly; sky blue, viral transcription; pink, RINA binding; yellow, ribosome localized; green, ribosomal subunit; purple, cytosolic large ribosomal subunit. The pink and black edges represents experimentally determined and co-expressed, respectively. (**B** and **C)** represent the biological and molecular functions of these proteins.

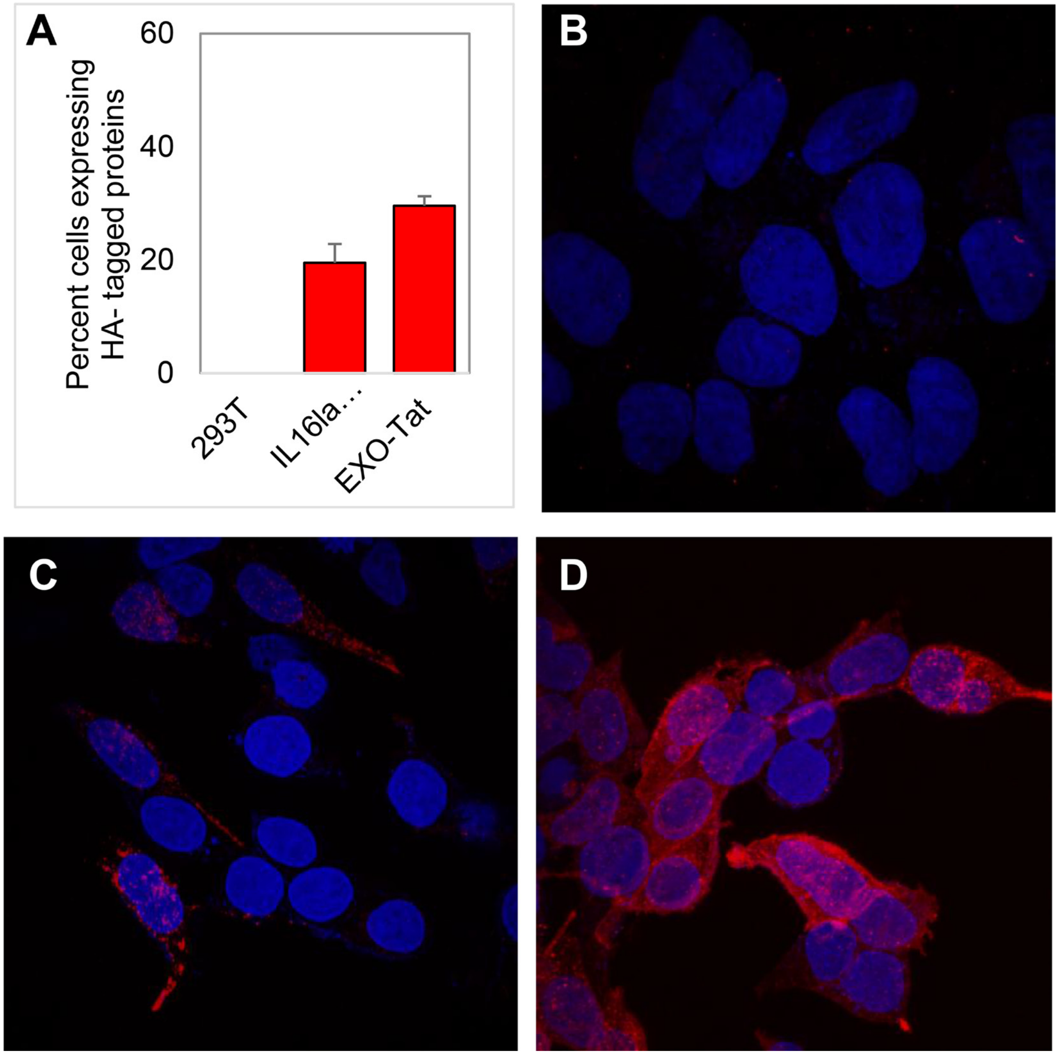

Supplementary Figure 4: The percent cells expressing HA-tagged proteins in the transfected cell lines. The population of 293T, IL16lamp2b and EXO-Tat cells were prepared and probed with HA antibodies against HA-tagged IL16lamp2b or EXO-Tat proteins. Positive staining was defined through intensity thresholding and percent HA positive cells determined by 15 fields per slide.

Supplementary Table 1: 293T cells-R1. See Supplementary Table 1

Supplementary Table 2: 293T cells-R2. See Supplementary Table 2

Supplementary Table 3: 293T cells-R3. See Supplementary Table 3

Supplementary Table 4: 293T cells-R4. See Supplementary Table 4

Supplementary Table 5: IL6 Lamp2B-R1. See Supplementary Table 5

Supplementary Table 6: IL6 Lamp2B-R2. See Supplementary Table 6

Supplementary Table 7: IL6 Lamp2B-R3. See Supplementary Table 7

Supplementary Table 8: IL6 Lamp2B-R4. See Supplementary Table 8

Supplementary Table 9: mExo-Tat-R1. See Supplementary Table 9

Supplementary Table 10: mExo-Tat-R2. See Supplementary Table 10

Supplementary Table 11: mExo-Tat-R3. See Supplementary Table 11

Supplementary Table 12: mExo-Tat-R4. See Supplementary Table 12

Supplementary Table 13: List of total peptides/proteins identified from the cell samples

Sample name	Identified unique peptides	Identified unique proteins	Total No of unique peptides	Total No of unique proteins
293T cells-R1	6449	2133	10386	2942
293T cells-R2	6265	2079		
293T cells-R3	6372	2156		
293T cells-R4	6343	2144		
IL16lamp 2b cells-R1	5928	2009	9662	2787
IL16lamp 2b cells-R2	6226	2072		
IL16lamp 2b cells-R3	6072	1987		
IL16lamp 2b cells-R4	6126	2093		
mExo Tat cell-R1	6263	2114	11840	3201
mExo Tat cells-R2	6817	2259		
mExo Tat cells-R3	5949	2082		
mExo Tat cells-R4	5977	2060		

Supplementary Table 14: Quantitative analysis of all peptides/proteins identified and quantified from 293T, IL16lamp 2b, and mExo-Tat cells. See Supplementary Table 14
